# Colonization of the tsetse fly midgut with commensal *Kosakonia cowanii* Zambiae inhibits trypanosome infection establishment

**DOI:** 10.1371/journal.ppat.1007470

**Published:** 2019-02-28

**Authors:** Brian L. Weiss, Michele A. Maltz, Aurélien Vigneron, Yineng Wu, Katharine S. Walter, Michelle B. O’Neill, Jingwen Wang, Serap Aksoy

**Affiliations:** 1 Yale School of Public Health, Department of Epidemiology of Microbial Diseases, New Haven, Connecticut, United States of America; 2 Southern Connecticut State University, New Haven, Connecticut, United States of America; Pennsylvania State University, UNITED STATES

## Abstract

Tsetse flies (*Glossina* spp.) vector pathogenic trypanosomes (*Trypanosoma* spp.) in sub-Saharan Africa. These parasites cause human and animal African trypanosomiases, which are debilitating diseases that inflict an enormous socio-economic burden on inhabitants of endemic regions. Current disease control strategies rely primarily on treating infected animals and reducing tsetse population densities. However, relevant programs are costly, labor intensive and difficult to sustain. As such, novel strategies aimed at reducing tsetse vector competence require development. Herein we investigated whether *Kosakonia cowanii* Zambiae (*Kco_Z*), which confers *Anopheles gambiae* with resistance to *Plasmodium*, is able to colonize tsetse and induce a trypanosome refractory phenotype in the fly. *Kco_Z* established stable infections in tsetse’s gut and exhibited no adverse effect on the fly’s survival. Flies with established *Kco_Z* infections in their gut were significantly more refractory to infection with two distinct trypanosome species (*T*. *congolense*, 6% infection; *T*. *brucei*, 32% infection) than were age-matched flies that did not house the exogenous bacterium (*T*. *congolense*, 36% infected; *T*. *brucei*, 70% infected). Additionally, 52% of *Kco_Z* colonized tsetse survived infection with entomopathogenic *Serratia* marcescens, compared with only 9% of their wild-type counterparts. These parasite and pathogen refractory phenotypes result from the fact that *Kco_Z* acidifies tsetse’s midgut environment, which inhibits trypanosome and *Serratia* growth and thus infection establishment. Finally, we determined that *Kco_Z* infection does not impact the fecundity of male or female tsetse, nor the ability of male flies to compete with their wild-type counterparts for mates. We propose that *Kco_Z* could be used as one component of an integrated strategy aimed at reducing the ability of tsetse to transmit pathogenic trypanosomes.

## Author summary

Tsetse flies transmit pathogenic African trypanosomes, which are the causative agents of socio-economically devastating human and animal African trypanosomiases. These diseases are currently controlled in large part by reducing the population size of tsetse vectors through the use of insecticides, traps and sterile insect technique. However, logistic and monetary hurdles often preclude the prolonged application of procedures necessary to maintain these control programs. Thus, novel strategies, including those aimed at sustainably reducing the ability of tsetse to transmit trypanosomes, are presently under development. Herein we stably colonize tsetse flies with a bacterium (*Kosakonia cowanii* Zambiae, *Kco_Z*) that acidifies their midgut, thus rendering the environment inhospitable to infection with two distinct, epidemiologically important trypanosome strains as well as an entomopathogenic bacteria. In addition to inducing a trypanosome refractory phenotype, colonization of tsetse with *Kco_Z* exerts only a modest fitness cost on the fly. Taken together, these findings suggest that *Kco_Z* could be applied to enhance the effectiveness of currently employed tsetse control programs.

## Introduction

Insects transmit numerous vertebrate pathogens that cause devastating disease throughout tropical and subtropical regions around the globe. The lack of effective and affordable vaccines, coupled with insect and pathogen resistance to pesticides and drug treatments, respectively, severely limits disease control. Many vertebrate pathogens are acquired by insect vectors via the ingestion of an infectious blood meal. The disease causing agents must then establish an infection in the insect’s gut prior to being transmitted to a new vertebrate host during a subsequent bite. In most cases pathogens are eliminated from the insect vector prior to transmission to a new vertebrate host. This outcome reflects the presence of dynamic active and passive immune barriers that function locally in the insect gut and systemically in the hemocoel [1–3].

Although few insect vectors support transmissible infections with vertebrate pathogens, all house symbiotic microorganisms in their gut that influence numerous aspects of their host’s physiological homeostasis. Symbiotic associations between arthropod disease vectors and enteric bacteria have been particularly well-studied in an effort to determine how these microbes influence their host’s ability to transmit disease [4–8]. Tsetse flies, which are the prominent vectors of pathogenic African trypanosomes, house a taxonomically diverse enteric microbiota that includes endosymbiotic *Wigglesworthia* and *Sodalis* [9] as well as an assemblage of bacteria obtained from the fly’s environment [10–12]. Both *Wigglesworthia* and *Sodalis* are maternally transmitted to developing intrauterine larvae during tsetse’s unique mode of viviparous reproduction [9,13]. *Wigglesworthia* influences trypanosome infection establishment in tsetse by regulating the production of trypanocidal PGRP-LB [14,15]. Additionally, tsetse that undergo larval development in the absence of this bacterium fail to synthesize a gut-associated peritrophic matrix during adulthood [16]. This structure is an important mediator of tsetse’s vector competence because it serves as a physical barrier that ingested parasites must traverse in order to successfully colonize the fly’s gut [17] and subsequently the salivary glands for transmission in saliva [18]. *Sodalis’* impact on tsetse vector competency is less known, although studies suggest that a positive correlation exists between the prevalence and density of this bacterium and trypanosome infection prevalence [19–23]. Like mosquitoes, tsetse’s gut also harbors a diverse population of bacteria obtained from the fly’s environment [10–12]. However, the effect of these bacteria on tsetse vector competency is poorly understood.

Mosquitoes, including *Anopheles gambiae* and *Aedes aegypti*, also house bacteria in their gut, and these microbes play a significant role in the ability of their host to transmit vertebrate pathogens. Boissiere et al. [24] discovered a positive correlation between the density of enteric Enterobacteriaceae and *Plasmodium* infection prevalence in field-captured *An*. *gambiae*. These midgut microbes, as well as the enteric microbiota found in *Ae*. *aegypti*, indirectly regulate their host’s vector competency by modulating basal expression of genes that encode anti-*Plasmodium* and anti-dengue effector proteins [5,25,26]. Other members of the mosquito enteric microbiota exert direct effects on their host’s vector competency. Specifically, a *Chromobacterium* isolated from *Ae*. *aegypti* secretes factors that exhibit anti-*Plasmodium* and anti-Dengue activity [27]. Also, laboratory reared *A*. *gambiae* present an abnormal *Plasmodium* refractory phenotype when their guts are colonized with *Kosakonia cowanii* Zambiae, (*Kco_Z*; previously designated *Enterobacter* sp Zambiae, *Esp_Z* [28]) that had been isolated from field-captured mosquitoes. *Kco_Z* was determined to produce reactive oxygen intermediates (ROIs) that exhibit direct anti-*Plasmodium* properties [29,30].

In this study we investigated whether *Kco_Z* isolated from *A*. *gambiae* is able to successfully colonize tsetse’s gut and induce parasite and pathogen refractory phenotypes in the fly. We found that this bacterium can reside stably in tsetse’s midgut without imparting a detrimental fitness cost on the fly. *Kco_Z* colonized tsetse present an acidified midgut environment that is inhospitable to both African trypanosomes and entomopathogenic *Serratia marcescens*. We discuss the potential utility of *Kco_Z* as a novel component of currently used area wide integrated pest management strategies aimed at controlling tsetse populations and thus transmission of African trypanosomes.

## Results

### Bacterial infection outcomes in tsetse’s midgut, and subsequent fly survival

**Fig 1:**
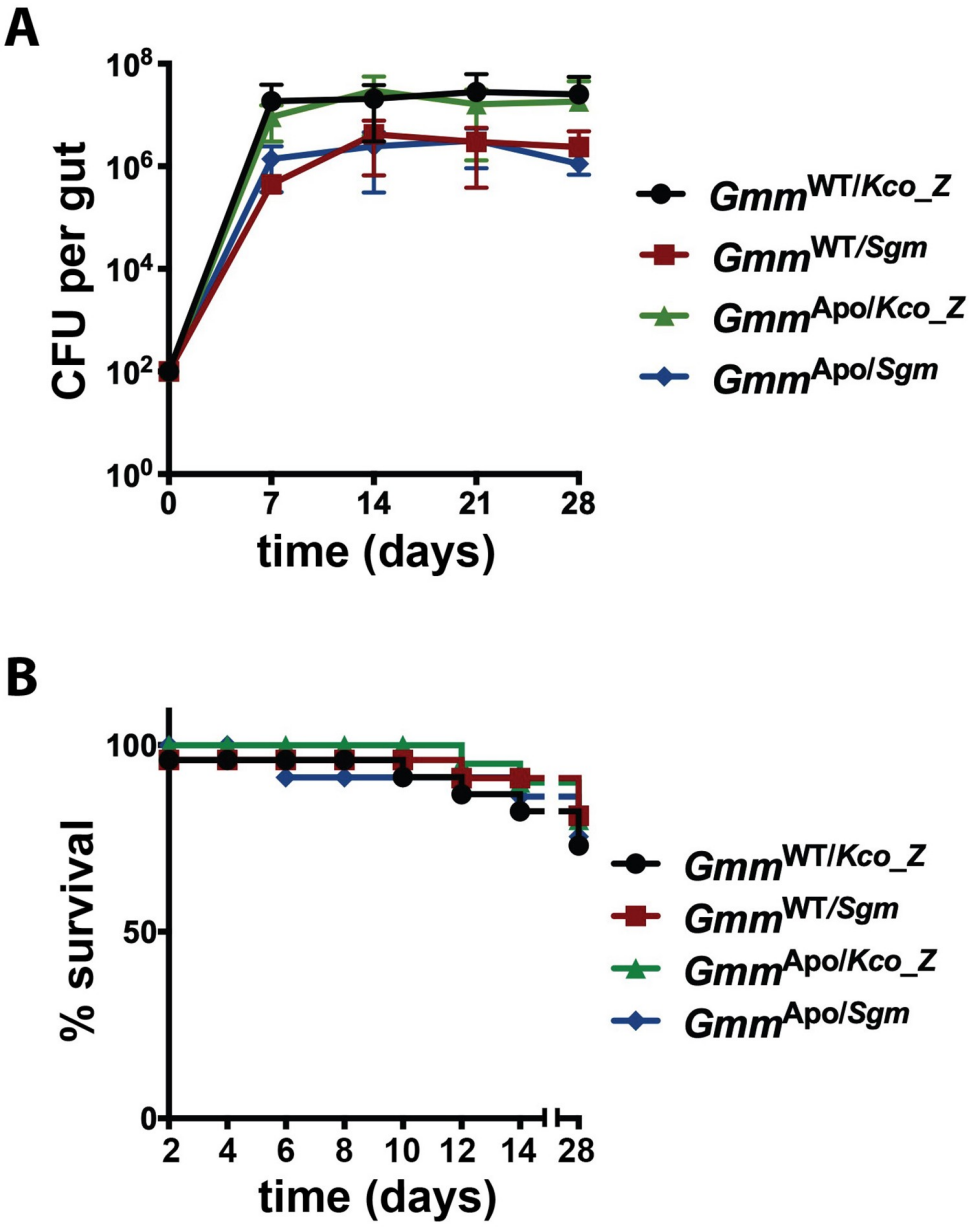
Bacterial colonization of tsetse’s midgut, and the effect on fly survival. Distinct groups of newly emerged adult wild-type (*Gmm*^WT^) and aposymbiotic (*Gmm*^Apo^) females were colonized with 1x10^3^ CFU of either *Kosakonia cowanii* Zambiae (*Kco_Z*) or *Sodalis* (*Sgm*), and then bacterial load and fly survival was measured. (A) Average number (±SEM) of bacterial CFUs per tsetse gut per group per time point. *n*≥5 individuals per group per timepoint. (B) Kaplan-Meier plot depicting survival of *Gmm*^WT^ and *Gmm*^Apo^ females colonized with either *Kco_Z* or *Sgm*. Infection experiments were performed using three distinct biological replicates (*n* = 25 flies per replicate). No significant difference in survival was observed between any of the fly groups (*p* = 0.88; log-rank test).

We investigated the ability of *Kco_Z* and *Sodalis* (as a control) to colonize the gut of both wild-type (hereafter referred to as ‘*Gmm*^WT^’) and symbiont-free tsetse (aposymbiotic, hereafter referred to as ‘*Gmm*^Apo^’). *Gmm*^WT^ flies were used to interrogate the interaction between *Kco_Z* and the natural tsetse microbiota, while the use of *Gmm*^Apo^ individuals allowed us to correlate the presence of distinct, experimentally introduced bacterial taxa with specific fly phenotypes. We challenged all flies *per os* with 1x10^3^ CFU of either *Kco_Z* or *Sodalis* in their first blood meal and then monitored bacterial proliferation over a 28 day period. By 7 days post-inoculation, midgut bacterial load in *Gmm*^WT^ flies that housed *Kco_Z* (*Gmm*^WT/*Kco_Z*^) and *Sodalis* (*Gmm*^WT/*Sgm*^) was 1.9x10^7^ ± 6.4x10^6^ CFU and 4.5x10^5^ ± 6.4x10^6^ CFU, respectively, and *Gmm*^Apo/*Kco_Z*^ (9.3x10^6^ ± 5.3x10^5^ CFU) and *Gmm*^Apo/*Sgm*^ (1.4x10^6^ ± 3.9x10^5^ CFU) flies harbored a similar bacterial load at the same time point post-inoculation (Fig 1A). The midgut load of *Kco_Z* and *Sodalis* did not change significantly in any of the fly groups over the following 21 days (Fig 1A), thus suggesting that the bacteria had achieved stable-state infections within their fly hosts by one week post-acquisition.

We next examined whether midgut infections with *Kco_Z* or *Sodalis* impacted tsetse survival. We found that 76% of *Gmm*^WT/*Kco_Z*^ individuals, and 84% of *Gmm*^WT/*Sgm*^ individuals, survived for 28 days following bacterial inoculation. Similarly, 84% and 80% of *Gmm*^Apo/*Kco_Z*^ and *Gmm*^Apo/*Sgm*^ flies, respectively, survived the duration of the experiment (Fig 1B). Percent survival was not significantly different between any of these groups, indicating that *Kco_Z* and *Sodalis* both exhibit commensal phenotypes in wild-type and aposymbiotic tsetse.

### *Kco_Z* is resistant to Peptidoglycan Recognition Protein-LB (PGRP-LB)

**Fig 2:**
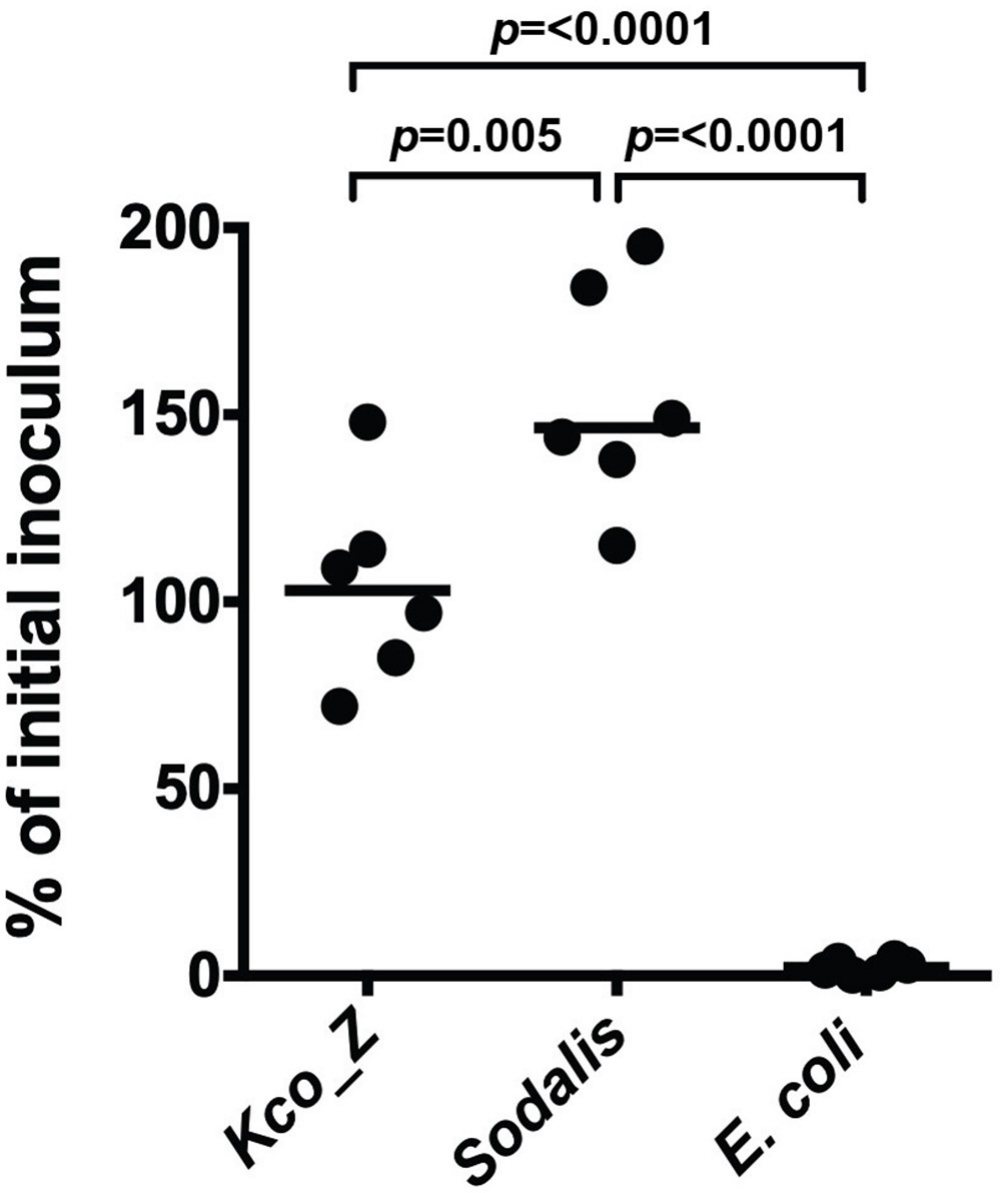
*Kco_Z* is resistant to normally bactericidal Peptidoglycan Recognition Protein-LB. Survival of cultured *Kco_Z*, *Sodalis* and *E*. *coli* following exposure (1 hr for *Kco_Z* and *E*. *coli*, and 24 hr for *Sodalis*) to recombinant (rec) PGRP-LB (10 μg/ml). Results are presented as % of initial inoculum, which was determined by dividing the number of bacterial CFU present after treatment with recPGRP-LB by the number of CFU present prior to inoculation. Each point on the graph represents a distinct bacterial culture. Statistical significance was determined using a one-way ANOVA followed by Tukey’s HSD post-hoc analysis.

The midgut of adult tsetse expresses *peptidoglycan recognition protein LB* (*pgrp-lb*), which encodes a pattern recognition receptor that exhibits potent antimicrobial activity [14,15]. Thus, in order to colonize tsetse’s midgut, a microorganism must be resistant to this molecule. We investigated whether innate resistance to PGRP-LB represents one mechanism that allows *Kco_Z* to colonize tsetse’s gut. We found that 108% (±16) of *Kco_Z* cells were able to survive 1 h in the presence of recPGRP-LB, while only 2.3% (±1.0) of *E*. *coli* cells survived for the same time period. Additionally, 154% (±14) of *Sodalis* cells survived following a 12 h incubation with recPGRP-LB (Fig 2). These findings suggest that like native *Sodalis*, *Kco_Z* is resistant to the antimicrobial properties of PGRP-LB and is able to survive in the presence of this protein (as indicated by a slight increase in bacterial load compared to the initial inoculate). This phenotype may facilitate this bacterium’s ability to successfully colonize tsetse’s gut.

### *Kco_Z* colonized aposymbiotic tsetse present a trypanosome refractory phenotype

**Fig 3:**
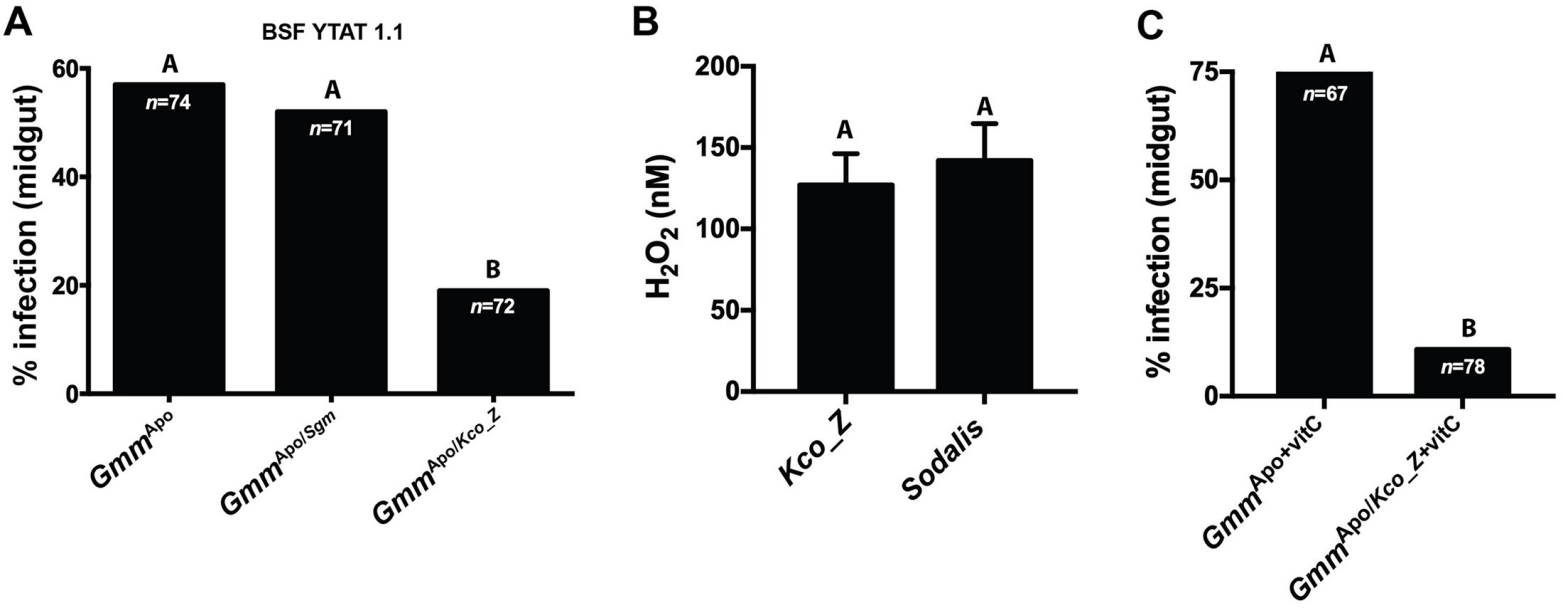
The trypanosome refractory phenotype exhibited by *Gmm*^Apo*/Kco_Z*^ flies is not directly caused by reactive oxygen intermediates. (A) Percentage of *Gmm*^Apo^, *Gmm*^Apo/*Sgm*^ and *Gmm*^Apo/*Kco_Z*^ flies harboring midgut infections with bloodstream form (BSF) YTAT 1.1 trypanosomes. Statistical analysis was performed using a GLM followed by multiple comparisons and Tukey contrasts, and different letters represents statistical significance between treatments and controls. (B) Mid-log phase cultures of *Kco_Z* and *Sodalis* synthesize similar quantities of H_2_O_2_ (*p* = 0.6; paired t-test). Measurements were taken from 7 distinct clonal populations of each bacterium. (C) Percentage of *Gmm*^Apo^ and *Gmm*^Apo/*Kco_Z*^ flies infected with BSF YTAT 1.1 trypanosomes after being fed blood meals containing antioxidant vitamin C over the course of the 14 day experiment. Despite exposure of both tsetse groups to the ROI-suppressing vitamin, *Gmm*^Apo/*Kco_Z*^ flies were still significantly more refractory to trypanosome infection than were *Gmm*^Apo^ individuals (*p* = 0.002; GLM Wald test). In (A), (B) and (C) different letters represents statistical significance between treatments and treatments and controls.

*Kco_Z* successfully colonizes the gut of *Gmm*^Apo^ flies, resides in the niche for at least 28 days, and has no impact on fly survival during that time period. Thus, we next evaluated whether colonization with this bacterium impacts trypanosome infection establishment in tsetse’s midgut. We began by challenging mature *Gmm*^Apo^ flies because they are highly susceptible to trypanosome infection (~50%) while their age-matched *Gmm*^WT^ counterparts are refractory (~3%) [16]. Distinct groups of eight day old *Gmm*^Apo/*Sgm*^ and *Gmm*^Apo/*Kco_Z*^ flies, which housed similar numbers of their respective exogenous bacteria (S1A Fig), were administered a meal supplemented with 1x10^6^ blood stream form (BSF) trypanosomes per ml of blood. Thereafter all flies were maintained on regular blood for two weeks, at which point their midguts were dissected and microscopically examined for the presence of parasites. An age-matched control cohort consisted of similarly challenged *Gmm*^Apo^ flies. We found that infection prevalence in the *Gmm*^Apo/*Sgm*^ group (57%) was similar to that of *Gmm*^Apo^ controls (52%), while infection prevalence in *Gmm*^Apo/*Kco_Z*^ individuals was significantly lower (19%) (Fig 3A). These data indicate that the presence of *Kco_Z* in tsetse’s gut interferes with the ability of trypanosomes to establish an infection in this niche. This parasite resistant phenotype is similar to that which occurs in the gut of *Kco_Z* colonized mosquitoes following exposure to malaria parasites [29,30].

### African trypanosomes are not susceptible to *Kco_Z* generated reactive oxygen intermediates

In *A*. *gambiae*, *Kco_Z* produces reactive oxygen intermediates (ROIs) that are directly toxic to *Plasmodium* [29,30]. ROIs have also been implicated as mediators of trypanosome infection outcomes in tsetse. Specifically, tsetse are rendered susceptible to trypanosome infection when the initial infectious blood meal is supplemented with the antioxidants vitamin C or cysteine [18,31]. These antioxidants detoxify ROIs that otherwise induce programmed cell death processes in trypanosomes [32]. In light of this information, we investigated the correlation between *Kco_Z* generated ROIs and the trypanosome refractory phenotypes we observed in adult *Gmm*^Apo/*Kco_Z*^ individuals. As an indicator of bacterial ROI production, we quantified H_2_O_2_ concentrations in supernatants from mid-log phase *Kco_Z* and *Sodalis* cultures. *Kco_Z* and *Sodalis* supernatants contained 127nM (±15) and 142nM (±13) of H_2_O_2_, respectively (Fig 3B).

We next tested whether ROIs produced by *Kco_Z* inhibit the ability of trypanosome to infect *Gmm*^Apo/*Kco_Z*^ flies. Individual groups of eight day old *Gmm*^Apo^ and *Gmm*^Apo/*Kco_Z*^ individuals were offered a blood meal containing infectious trypanosomes together with the antioxidant vitamin C. All trypanosome challenged flies were subsequently maintained on vitamin C supplemented blood for 14 days. Under these conditions, 74% of *Gmm*^Apo+vitC^ flies were infected with trypanosomes, while only 11% of their *Gmm*^Apo/*Kco_Z*+vitC^ counterparts housed parasite infections (Fig 3C). These results suggest that ROIs produced by *Kco_Z* that reside stably in tsetse’s gut are not the sole determinants of the fly’s susceptibility to infection with trypanosomes.

### *Kco_Z* produces acid that is toxic to trypanosomes

**Fig 4:**
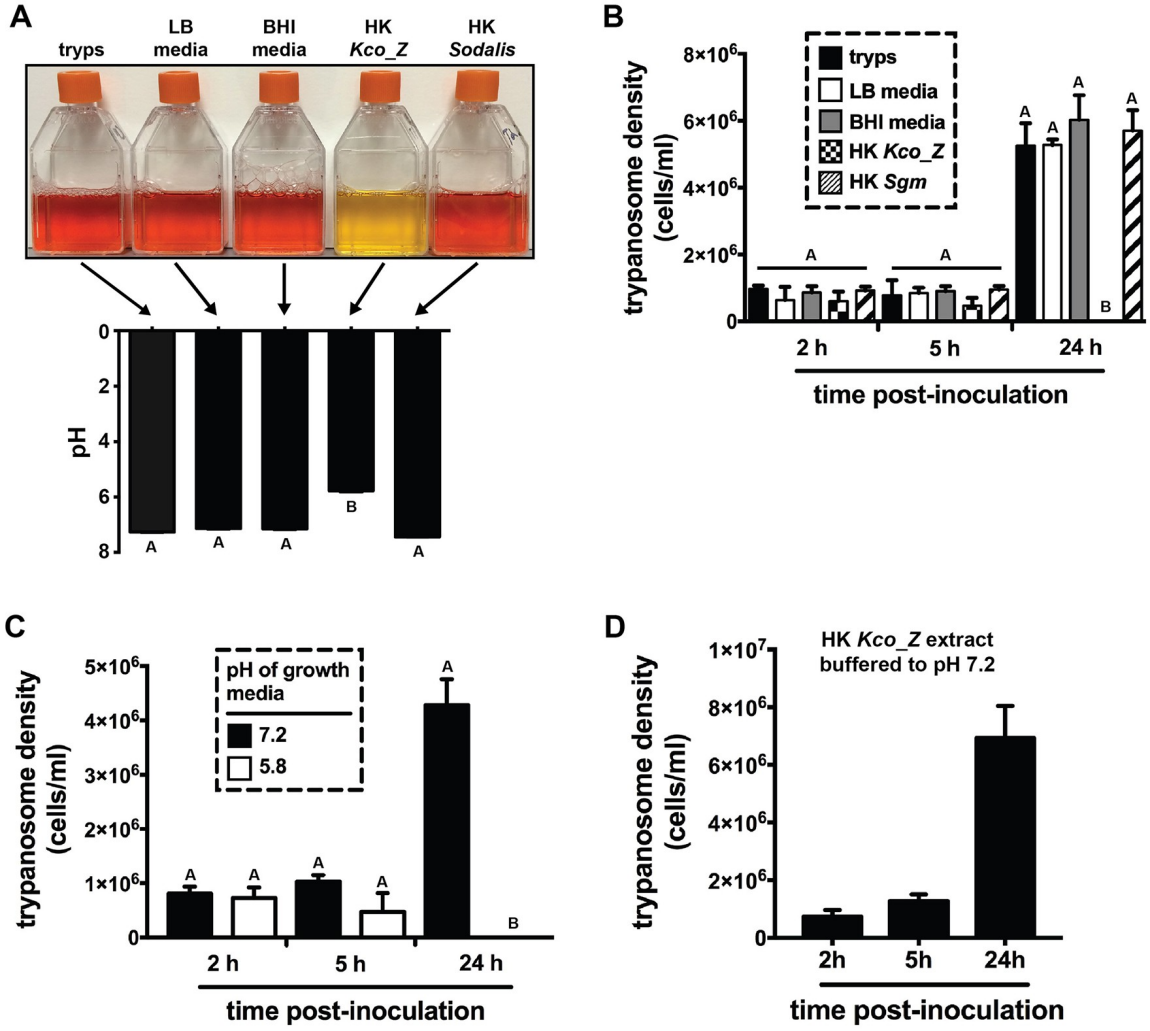
*Kco_Z* produces a low pH environment that is toxic to trypanosomes. (A) Early log phase trypanosomes (*T*. *b*. *brucei* YTAT 1.1), cultured in 10ml of Beck’s medium containing the pH sensitive dye phenol red, exposed to 1ml of heat treated LB media (*Kco_Z* culture medium), 1ml of heat treated BHI media (*Sodalis* culture medium), heat killed (HK) *Kco_Z* (5x10^6^ cells) in 1ml of LB media and HK *Sodalis* (5x10^6^ cells) in 1ml of BHI media. Controls are trypanosomes alone (tryps). All heated treatments and controls were allowed to cool to room temperature prior to adding them to the trypanosome cultures. Two hours post-treatment, culture pH was measured. HK *Kco_Z* significantly reduced the pH of the trypanosome culture (*p*<0.001). The experiment was repeated using 6 distinct clonal trypanosome populations (the image represents one of the six replicates). (B) Density of trypanosomes in culture 2h, 5h and 24h after addition of the treatments described in (A) above. At the 24h time point, all trypanosomes exposed to HK *Kco_Z* extracts were dead while those from the other groups were replicating similarly to controls. (C) Density of trypanosomes cultured in normal (pH 7.2) and artificially produced (via the addition of 0.1N HCl) acidic (pH 5.8) environments. Artificial acidic conditions kill all trypanosomes with 24 h. (D) The density of cultured trypanosomes exposed to HK *Kco_Z* extracts buffered to pH 7.2 (via the addition of 0.1N NaOH). The buffering treatment rescues parasite growth. In (A), (B) and (C), statistical significance was determined using a one-way ANOVA followed by Tukey’s HSD post-hoc analysis in (A), and a two-way ANOVA followed by Tukey’s HSD post-hoc analysis in (B) and (C). Different letters represent statistical significance between treatments and controls. In (B), (C) and (D), experiments were performed using 5 or 6 distinct clonal trypanosome populations.

We observed that *Gmm*^Apo/*Kco_Z*^ flies are significantly more refractory to infection with trypanosomes than are *Gmm*^Apo/*Sgm*^ individuals, despite the fact that *Sodalis* and *Kco_Z* produce similar amounts of H_2_O_2_. This outcome implies that *Kco_Z* modulates trypanosome infection outcomes in tsetse via a mechanism other than ROI production. Members of the genus *Kosakonia* [33–35], as well as several enteric commensals including *Enterobacter* spp. [36–38], produce organic acids, and these products can inhibit pathogen growth by creating an acidic environment [39,40]. Because many trypanosomatids, including members of the genera *Trypanosoma* and *Leishmania*, are highly sensitive to environmental pH [41,42], we investigated whether *Kco_Z* creates an acidic environment that prohibits *T*. *brucei* growth *in vitro*. Specifically, we heat killed (HK) *Kco_Z* (1x10^6^ log-phase in 500 μl of LB media) and added the solution to trypanosome cultures maintained *in vitro*. This medium includes phenol red, which is a pH-sensitive dye that when in solution turns from red-pink to yellow as the quantity of acid in the environment increases. Addition of this HK *Kco_Z* extract immediately turned the Beck’s media yellow, and the pH measured at 5.8 (± 0.39). This value was significantly lower than trypanosome cultures that were supplemented with 500 μl of 1x10^6^ log-phase HK trypanosomes (pH 7.3 ± 0.28), HK *Sodalis* (pH 7.4 ± 0.39) or LB (*Kco_Z* growth media; pH 7.1 ± 0.28) or MM media (*Sodalis* growth media; pH 7.2± 0.29) alone (Fig 4A). We subsequently monitored trypanosome growth in cultures that received the above-mentioned supplements. We observed that trypanosomes failed to replicate in Beck’s medium that contained HK *Kco_Z* extracts, while trypanosomes multiplied in all of the other culture conditions (Fig 4B).

Heat-killed *Kco_Z* extracts create an acidic environment when added to trypanosome cultures, and trypanosomes fail to replicate in this environment. These findings do not rule out the possibility that trypanosomes are capable of surviving *Kco_Z*-induced acidic conditions, and instead, some other unknown component of the medium [e.g., a bacterium-derived trypanocidal molecule(s)] exhibits toxic properties. To address this possibility, we monitored trypanosome growth in Beck’s medium, the pH of which was artificially decreased to 5.8 (the same as that achieved by adding HK *Kco_Z* extracts) via the addition of exogenous acid. Under these conditions trypanosomes failed to replicate (Fig 4C). Furthermore, when we buffered Beck’s medium containing HK *Kco_Z* extracts back up to pH 7.2, trypanosomes replicated normally (Fig 4D). Taken together, these data indicate that *Kco_Z* produces an acidic environment that is toxic to trypanosomes, thus impeding their growth *in vitro*.

### *Kco_Z* acidifies tsetse’s gut

**Fig 5:**
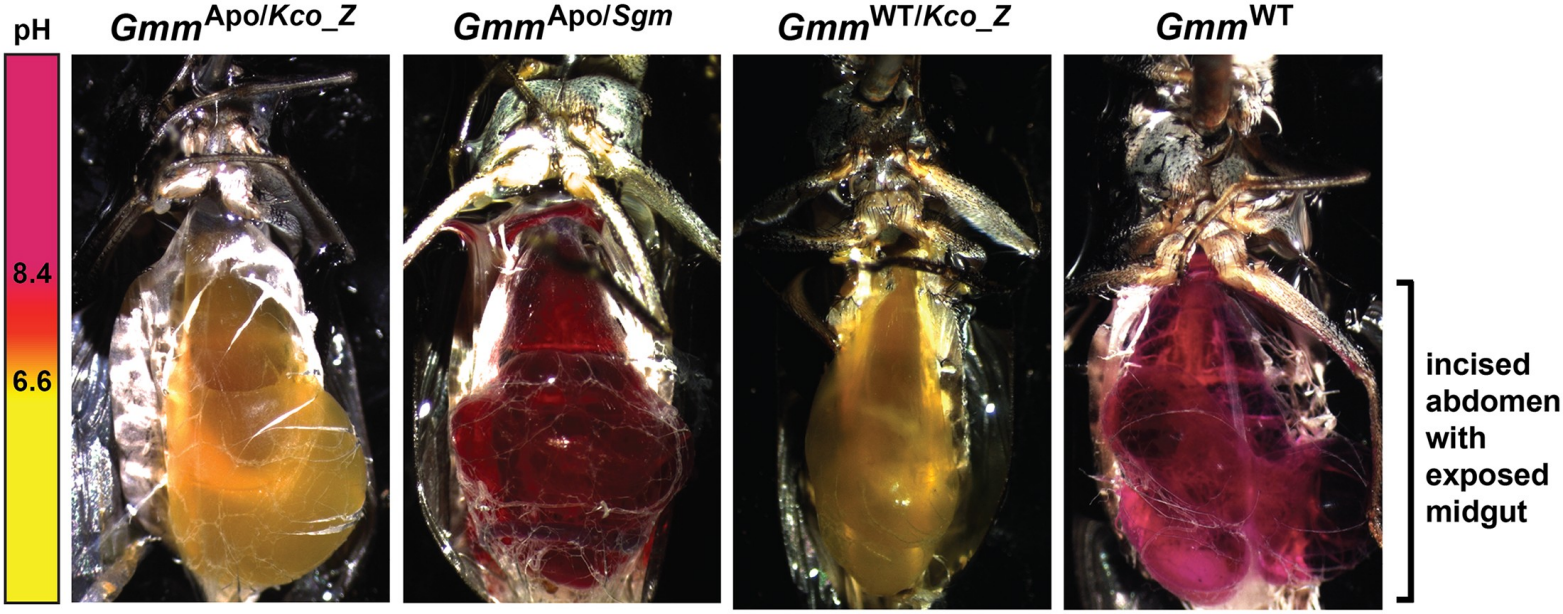
*Kco_Z* acidifies tsetse’s gut. Midgut pH of *Gmm*^Apo/*Kco_Z*^, *Gmm*^Apo/*Sgm*^, *Gmm*^WT/*Kco_Z*^ and *Gmm*^WT^ flies. Distinct groups of teneral *Gmm*^Apo^ flies were inoculated *per os* with either *Kco_Z* or *Sodalis*, while *Gmm*^WT^ individuals received *Kco_Z* (all flies received 5x10^4^ CFU of bacteria per ml of blood) or no bacteria. Five days post-inoculation, all individuals were offered a meal containing sucrose (2.5%) and phenol red (0.04%) dissolved in water. Twenty-four hours later, fly abdomens were excised and the color of the solution found within the midgut was observed. Each image represents one of five flies monitored for each treatment.

We observed that trypanosomes are unable to multiply when cultured in medium supplemented with acidic *Kco_Z* extracts. Thus, we next investigated whether *Kco_Z* produces acid *in vivo* in tsetse’s gut. To do so we colonized teneral, aposymbiotic flies with either *Kco_Z* or *Sodalis*, and 5 days later fed them a meal containing 2.5% sucrose and 0.04% phenol red solubilized in water. Twenty-four hours later, midguts from a sample of flies (*n* = 8 per group) were excised and plated on solid medium containing phenol red. *Gmm*^Apo/*Kco_Z*^ and *Gmm*^Apo/*Sgm*^ flies housed similar densities of the introduced bacteria (S1B Fig), and their respective mediums changed color to reflect corresponding pH shifts (S1C Fig). The remaining flies were dissected to expose their midgut *in situ*, and the color of the gut contents was visualized microscopically. We observed that the gut contents of *Gmm*^Apo/*Kco_Z*^ flies were yellow in color (Fig 5), thus indicating that the environment had become acidified. Conversely, the gut contents of *Gmm*^Apo/*Sgm*^ individuals were red, which is similar to the more alkaline environment present in the gut of *Gmm*^WT^ tsetse (Fig 5).

Finally, we investigated whether *Kco_Z* also produces acid in the gut *Gmm*^WT^ tsetse by inoculating teneral individuals with 1x10^3^ CFU of the bacterium (these flies were designated *Gmm*^WT/*Kco_Z*^). Five days later a cohort of *Gmm*^WT/*Kco_Z*^ females (these flies housed 1.27x10^6^ ± 8.6x10^4^ *Kco_Z* at this time point; S1D Fig), as well as age matched *Gmm*^WT^ controls, were fed a sugar meal containing phenol red (as described above) to observe gut pH. Similar to our results noted in *Gmm*^Apo/*Kco_Z*^ flies, we observed that the gut of *Gmm*^WT/*Kco_Z*^ individuals was yellow, thus indicative of an acidified environment. Conversely, the gut environment of *Gmm*^WT^ flies was red and thus comparatively alkaline (Fig 5). Thus, the presence of indigenous symbionts does not impede the ability of *Kco_Z* to acidify the gut of wild-type flies.

### *Gmm*^WT/Kco_Z^ are highly refractory to infection with trypanosomes and entomopathogenic bacteria

**Fig 6:**
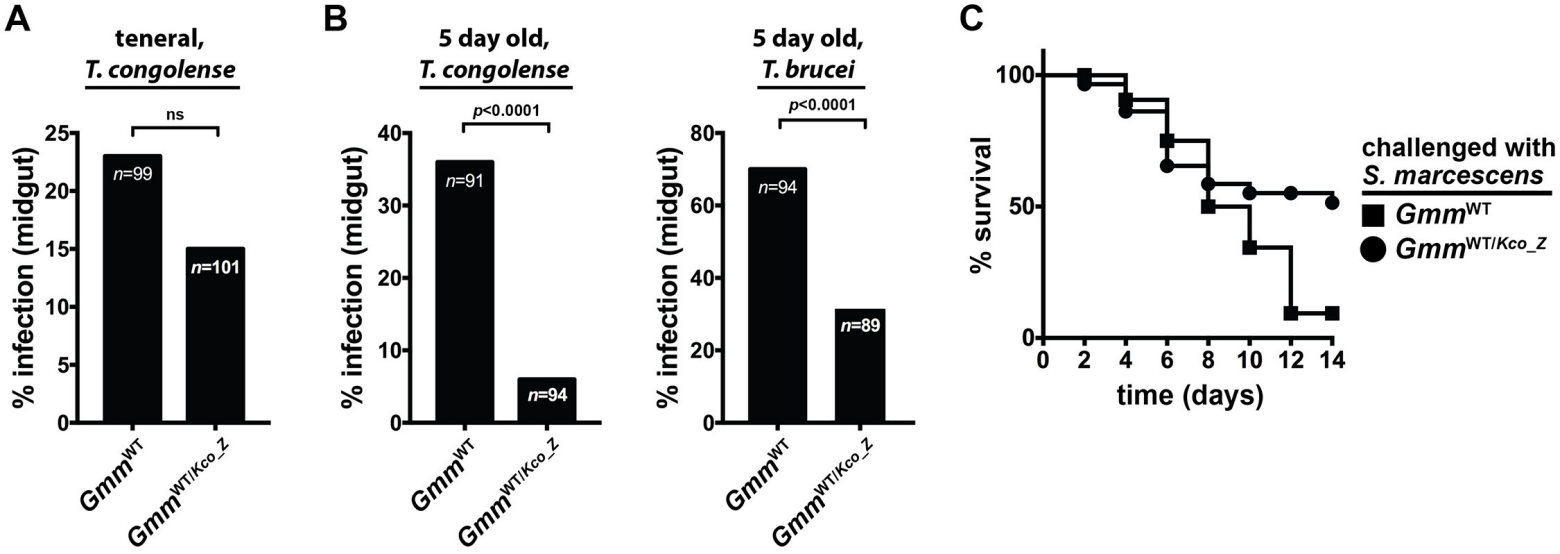
*Gmm*^WT/*Kco_Z*^ flies are significantly more refractory to infection with parasitic trypanosomes and entomopathogenic *S*. *marcescens*. (A) Percentage of *Gmm*^WT^ and *Gmm*^WT/*Kco_Z*^ flies infected with *T*. *congolense* 14 days after they were co-inoculated with *Kco_Z* and parasites in their first (teneral) blood meal. (B) Percentage of *Gmm*^WT^ and *Gmm*^WT/*Kco_Z*^ flies infected with *T*. *congolense* (left graph) and *T*. *brucei* (right graph). For these experiments *Gmm*^WT/*Kco_Z*^ flies housed the exogenous bacteria for five days prior to challenge with trypanosomes. In (A) and (B) Statistical analysis was performed using a GLM followed by multiple comparisons and Tukey contrasts, and different letters represents statistical significance between treatments and controls. (C) Kaplan-Meier plot depicting survival of *Gmm*^WT^ and *Gmm*^WT/*Kco_Z*^ flies following challenge (three days after administering *Kco_Z* or *Sodalis*) with *S*. *marcescens*. Infection experiments were performed using three distinct biological replicates (*n* = 25 flies per replicate). *Gmm*^WT/*Kco_Z*^ flies were also significantly more refractory to *S*. *marcescens* infection than were their *Gmm*^WT^ counterparts (*p* = 0.001; log-rank test).

We hypothesized that exogenous microorganisms would be unable to successfully infect *Gmm*^WT/*Kco_Z*^ due to their acidified midgut environment. To test this hypothesis we first co-inoculated teneral *Gmm*^WT^ males with *Kco_Z* and *T*. *congolense* parasites. Two weeks post-challenge we observed no significant difference in the percentage of *Gmm*^WT/*Kco_Z*^ (15%) and control *Gmm*^WT^ (23%) flies that harbored trypanosome infections in their midguts (Fig 6A). We next inoculated teneral *Gmm*^WT^ males with *Kco_Z* and then three days later (5 day old adults) challenged *Gmm*^WT/*Kco_Z*^ individuals with either *T*. *congolense* or *T*. *brucei* parasites (both of these parasite species are naturally transmitted by *G*. *m*. *morsitans*; [43,44]). Under these conditions we observed that *Gmm*^WT/*Kco_Z*^ males were significantly more refractory to infection with both parasite species (*T*. *congolense*, 6%; *T*. *brucei*, 32%) than were their age-matched *Gmm*^WT^ counterparts (*T*. *congolense*, 36%; *T*. *brucei*, 70%; Fig 6B). Thus, tsetse must house an established *Kco_Z* infection in its gut at the time of trypanosome challenge in order to present a refractory phenotype. Finally, we found that trypanosome infected *Gmm*^WT/*Kco_Z*^ house similar densities of *Kco_Z* as do age-matched individuals that eliminated their trypanosome infection (S1E Fig), again indicating that exogenous *Kco_Z* appears to be resistant to tsetse’s trypanocidal immune response. Additionally, the presence of tsetse’s indigenous, enteric microbiota does not interfere with *Kco_Z* mediated obstruction of trypanosome infection establishment.

Finally, we investigated whether *Kco_Z* also protects tsetse against infection with an entomopathogenic bacteria. To do so teneral *Gmm*^WT^ males were fed 1x10^3^ CFU of *Kco_Z*, and then three days later, the same dose of *Serratia marcescens* strain db11, which is highly virulent to wild-type tsetse [17,18,45]. Five day old *Gmm*^WT^ males infected with the same dose of *S*. *marcescens* were used as controls. Fly survival following *Serratia* inoculation was monitored over a 14 day period in both fly groups. We observed that 51% and 9% of *Gmm*^WT/*Kco_Z*^ and *Gmm*^WT^ individuals, respectively, survived their infection with *S*. *marcescens* (Fig 6C).

Taken together, our results detailed above indicate that wild-type tsetse present a parasite and entomopathogen refractory phenotype when they house an established *Kco_Z* infection in their gut. This phenotype like occurs because the acidified nature of the gut environment is inhospitable to the development of exogenous microbes.

### *Kco_Z* infection exerts a minimal fitness cost on tsetse

*Kco_Z* produces acid in tsetse’s gut such that the environment becomes inhospitable to trypanosomes. To address whether decreased midgut pH adversely impacts tsetse fitness, we quantified several fitness parameters in *Gmm*^WT/*Kco_Z*^ flies (a sample of these individuals housed 1.39x10^6^ ± 1.1x10^5^ *Kco_Z* at the time they were used for experimentation; S1F Fig).

**Fig 7:**
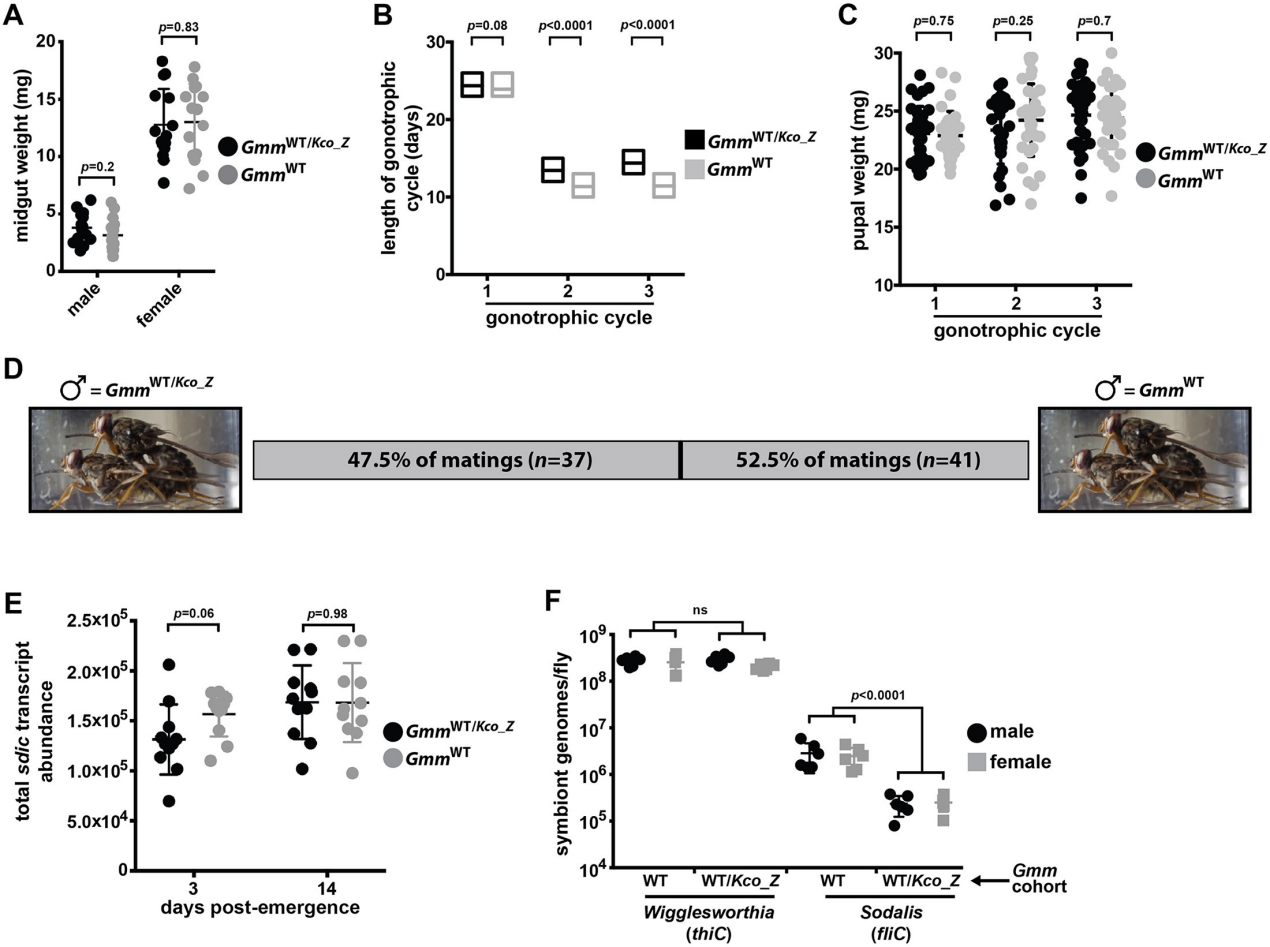
*Kco_Z* infection impacts specific tsetse fitness parameters. (A) Midgut weight, as an indicator of blood meal digestion proficiency, in six day old *Gmm*^WT/*Kco_Z*^ and *Gmm*^WT^ males and females (guts were weighed 24 h after the flies had consumed their last blood meal). Each point on the graph represents one individual, and statistical significance was determined via multiple t-tests. (B). Gonotrophic cycle (GC) length of *Gmm*^WT/*Kco_Z*^ and *Gmm*^WT^ females. Age-matched, pregnant females from each group (*n* = 35 per group) were housed in individuals cages and monitored daily to observe frequency of pupal deposition. Statistical significance was determined via log-rank test. (C) Weight of pupae from *Gmm*^WT/*Kco_Z*^ and *Gmm*^WT^ females over three GCs. Each point on the graph represents one individual, and statistical significance was determined via multiple t-tests. (D) Mating competitiveness of *Gmm*^WT/*Kco_Z*^ compared to *Gmm*^WT^ males. Matings were setup in individual cages (*n* = 80). Each cage housed a virgin female, to which one virgin *Gmm*^WT/*Kco_Z*^ and *Gmm*^WT^ male was added. Males and females were age-matched, and each had fed twice prior to exposure. Statistical significance was determined via Chi-squared test. (E) Sperm abundance in the reproductive tracts of three and 14 day old (fed twice) virgin *Gmm*^WT/*Kco_Z*^ and *Gmm*^WT^ males. Sperm quantity is a reflection of *sperm-specific dynein intermediate chain* (*sdic*) transcript abundance. Absolute *sdic* transcript abundance was determined by comparing experimental sample cycle threshold (C_t_) values to those derived from an *sdic* internal standard curve. Each point on the graph represents one individual, and statistical significance was determined via multiple t-tests. (F) Quantitation of *Wigglesworthia* and *Sodalis* in *Gmm*^WT/*Kco_Z*^ and *Gmm*^WT^ male and female tsetse. Abundance of symbiont specific gene transcripts (*Wigglesworthia*, *thiC*; *Sodalis*, *fliC*) was used as a proxy to quantify bacterial load. This was performed by comparing *thiC* and *fliC* cycle threshold (C_t_) values in *Gmm*^WT/*Kco_Z*^ and *Gmm*^WT^ flies to those derived from symbiont gene-specific internal standard curves. *Wigglesworthia* and *Sodalis* can be polyploid (Rio et al., 2006; Weiss et al., 2006), and as such, we normalized symbiont genome copy number to constitutively expressed tsetse *gapdh* copy number. Each point on the graph represents one individual, and statistical significance was determined via multiple t-tests.

We began by measuring midgut weight, which reflects over all digestive health. We observed no significant difference in midgut weight between 8 day old *Gmm*^WT/*Kco_Z*^ and *Gmm*^WT^ males (3.8 ± 1.1 mg and 3.2 ± 1.2 mg, respectively) and females (12.8 ± 1.8 mg and 13.0 ± 1.8 mg, respectively) 24 hrs after acquiring their last blood meal (Fig 7A). We next measured fecundity parameters in female and male *Gmm*^WT/*Kco_Z*^ and *Gmm*^WT^ flies to determine if stable infection with this bacterium would alter their reproductive capacity. We began by measuring gonotrophic cycle (GC) duration of *Gmm*^WT/*Kco_Z*^ and *Gmm*^WT^ females. The length of the 1^st^ GC was not significantly different between *Gmm*^WT/*Kco_Z*^ (24.0 ± 1.2 days) and *Gmm*^WT^ females (24.0 ± 0.9 days) (Fig 7B). However, the 2^nd^ and 3^rd^ GCs of *Gmm*^WT/*Kco_Z*^ females (13.0 ± 1.1 and 14.0 ± 1.1 days, respectively) were significantly longer than those of their age-matched WT counterparts (11.7 ± 1.0 and 11.5 ± 1.1 days, respectively) (Fig 7B). We also determined that pupal weight from all three GCs was similar between both fly groups (GC1, *Gmm*^WT/*Kco_Z*^ = 23.1 ± 1.5 mg, *Gmm*^WT^ = 22.9 ± 1.4 mg; GC2, *Gmm*^WT/*Kco_Z*^ = 23.4 ± 1.7 mg, *Gmm*^WT^ = 24.2 ± 1.8 mg; GC3, *Gmm*^WT/*Kco_Z*^ = 24.7 ± 1.7 mg, *Gmm*^WT^ = 24.4 ± 1.6 mg) (Fig 7C). Thus, *Kco_Z* infection impacts the reproductive physiology of female tsetse by increasing GC duration and hence the number of offspring infected individuals are able to produce over the course of their lifespan. However, despite this effect, infection with this bacterium does not impact pupal weight.

We also investigated the effect of *Kco_Z* infection on the reproductive fitness of male tsetse by comparing the mating competitiveness of *Gmm*^WT/*Kco_Z*^ and *Gmm*^WT^ individuals. To do so we set up 80 individual cages, each of which contained one sexually mature virgin female. We subsequently placed one sexually mature *Gmm*^WT/*Kco_Z*^ and *Gmm*^WT^ male in each cage and monitored the arena to determine which of the two males successfully mated with the female. We observed that 47.5% of matings occurred between *Gmm*^WT/*Kco_Z*^ males and females (neither male mated with the female in two of the cages) (Fig 7D), thus indicating that *Kco_Z* infection does not significantly alter male mating competitiveness (χ^2^ = 0.1025641, df = 1, *p* = 0.748774). Next we compared the number of sperm present in three and 14 day old *Gmm*^WT/*Kco_Z*^ and *Gmm*^WT^ males by quantifying transcript abundance of *sperm-specific dynein intermediate chain* (*sdic*). The *Drosophila* homologue of this gene is transcribed exclusively in sperm cells [46] and is used to quantify sperm abundance [47]. We observed no significant difference in *sdic* transcript abundance between three day old or 14 day old *Gmm*^WT/*Kco_Z*^ and *Gmm*^WT^ males (Fig 7E).

Finally, we examined whether the presence of *Kco_Z* impacts the load of endosymbiotic *Wigglesworthia* and *Sodalis*. These measurements, which were taken at 14 days post-inoculation with *Kco_Z*, are important because tsetse’s microbiota impact many aspects of their host’s fitness, including fecundity and immune system development and function [48,49]. We observed that infection with *Kco_Z* did not significantly alter the load of tsetse’s midgut (bacteriome) population of obligate *Wigglesworthia* in *Gmm*^WT/*Kco_Z*^ males or females (Fig 7F). Conversely, midguts from *Gmm*^WT/*Kco_Z*^ males and females housed significantly fewer *Sodalis* than did midguts from their *Gmm*^WT^ counterparts (Fig 7F). Taken together, these data indicate that *Kco_Z* significantly impacts specific fitness parameters in both female and male flies.

## Discussion

Morbidity and mortality caused by vector-borne diseases currently inflicts a devastating socioeconomic burden on a significant percentage of the global population. To reduce this burden, novel disease control strategies that inhibit pathogen maturation within arthropod disease vectors require development. The enteric microbiota is being increasingly studied for use in this context, and one such novel strategy could employ the use of ‘probiotic’ bacteria, the presence of which would alter the physiology of the vector’s gut to make the environment inhospitable to pathogens. Herein we use the tsetse fly model system to highlight how an exogenous bacterium can be employed in this capacity to impede infection establishment of two pathogens in this insect disease vector. Specifically, we determined that *Kco_Z*, which is a bacterium found naturally in the gut of some *An*. *gambiae* populations, and directly kills *Plasmodium* by producing anti-parasitic ROIs [29,30], can stably colonize tsetse’s gut for at least 28 days. When the bacterium is present in this niche, tsetse are significantly more refractory to infection with parasitic African trypanosomes and entomopathogenic *S*. *marcescens* than are flies that house only their indigenous microbiota. *Kco_Z* creates this inimical environment by acidifying tsetse’s gut such that trypanosomes and *S*. *marcescens*, which are sensitive to these conditions, are no longer able to successfully infect the fly. While infection with *Kco_Z* exerts only a negligible effect on tsetse’s reproductive fitness, the bacterium’s presence does reduce the load of endosymbiotic *Sodalis*. Cumulatively, our findings suggest that *Kco_Z* could be used in a natural setting to artificially reduce disease transmission by this arthropod vector.

Herein we demonstrate that *Kco_Z* is able to stably colonize tsetse’s gut, which is an outcome that likely results at least in part from the bacterium’s resistance to antimicrobial PGRP-LB. This protein is constitutively produced in the fly’s midgut and directly kills trypanosomes and *E*. *coli* K12 (Fig 2 in this study, and [16]). *Kco_Z* resistance to tsetse PGRP-LB may reflect one of many bacterial adaptations that result from residing within the immunologically hostile environment of the insect midgut. While the specific physiological mechanism(s) that *Kco_Z* uses to facilitate its colonization of tsetse’s midgut are currently unknown, the bacterium survives for prolonged periods within the gut of *An*. *gambiae* in part by increasing expression of genes that encode a type III secretion system apparatus protein as well as glutathione S-transferase and oxidoreductase [30]. Type III secretion system proteins can facilitate bacterial penetrance into host cells [50] and be involved in subversion of host immunity [51], while the latter two proteins are antioxidant pathway components that mediate redox homeostasis in oxidatively stressful environments such as the insect midgut [52,53]. *Kco_Z* may employ similar mechanisms to survive in tsetse’s immunologically hostile gut. *Sodalis* is also resistant to tsetse antimicrobial immune response [9,54], which may be the result of structural adaptations present in exposed bacterial surface coat molecules, including lipopolysaccharide [55] and outer membrane protein A [56]. Furthermore, *Sodalis* enters into host cells through the use of a type III secretion system [57], which may further protect the bacterium from tsetse’s immunologically hostile midgut environment. Likewise, similar mechanisms may facilitate *Kco_Z* survival in this niche. Additionally, *Kco_Z*, like *Sodalis* [58], may reside extracellularly in the endoperitrophic space of tsetse’s midgut. In this position, the fly’s peritrophic matrix barrier would physically separate the bacteria from immunocompetent epithelial cells, thus reducing their exposure to harmful antimicrobial responses.

Microbes can alter their environment such that it either favors or hinders its own prosperity as well as the prosperity of other resident organisms [59]. Depending on specific physiological circumstances, these effects can reflect the consumption of resources and/or the production of beneficial or harmful metabolic byproducts [60,61]. Tsetse’s sole energy source, vertebrate blood, is rich in glucose. Many bacterial taxa, including members of the Enterobacteriaceae (in which *Kosakonia* and *Enterobacter* spp. cluster), ferment this sugar, thus producing hydrogen that acidifies their environment (38,61]. The acidic environment present in tsetse’s gut when *Kco_Z* resides stably in the tissue likely results at least in part from the bacterium’s utilization of blood glucose as an energy source. The pH in the gut of insect vectors, including tsetse flies [62], sand flies [63] and mosquitoes [64] is normally alkaline, and the parasites they transmit, as well as other enteric microbes, are adapted to survive in this environment. Correspondingly, our results indicate that *Kco_Z* induced conditions in tsetse’s gut detrimentally impact not only trypanosomes but also other enteric microbes including entomopathogenic *S*. *marcescens* and symbiotic *Sodalis*. *Kco_Z* mediated suppression of *S*. *marcescens*, or any other pathogen, would have obvious benefits to the fly. However, dysbiosis of tsetse’s facultative and commensal enteric microbiota could impact the fly’s overall fitness and/or vector competency. For example, reducing *Sodalis* density significantly decreases tsetse longevity [20]. This may prove beneficial because flies with a reduced life span could perish before trypanosomes are able to complete their 20–30 extrinsic incubation period [65]. A reduction in *Sodalis* density could be further beneficial because tsetse that house relatively low densities of the bacterium are less likely to be infected with trypanosomes than are individuals that house more of the symbiont [19–23]. Thus, the trypanosome refractory phenotype presented by *Kco_Z* colonized tsetse may result in part from, or be enhanced by, the fact that they contain fewer *Sodalis*. Finally, the midgut of wild tsetse is colonized by a transient population of environmentally acquired bacteria [9]. The contribution of these bacteria to tsetse’s physiology has not been characterized, and as such, interference with this microbial population could further alter the fly’s physiological homeostasis. To the contrary, the environmentally acquired microbiota could out-compete *Kco_Z* that reside in tsetse’s gut, or could prevent the bacterium from acidifying the environment. Future studies are required to elucidate microbe-microbe interactions in the gut *Kco_Z* colonized flies after their release into the field.

Reducing the incidence of African trypanosomiases has to date been achieved largely by controlling the size of tsetse populations. This process is currently accomplished by employing area wide integrated pest management (AW-IPM) strategies that make use of insecticides, traps and sterile insect technique (SIT) [66,67]. SIT involves sequentially releasing a large number of sterilized males (achieved by irradiating pupae or adults) into the target environment. These males reproductively outcompete wild males for female mates, and the population size drops significantly, or the fly is completely eradicated [68]. The efficacy of SIT as a means of controlling tsetse populations is well exemplified on Unguja Island (the large island of the Zanzibar archipelago), where the technique was used to eradicate *G*. *austeni*, the main vector of trypanosomes that cause animal African trypanosomiasis in that locale [69]. One shortcoming of this procedure is that releasing large numbers of sterile males significantly increases the population of potential disease vectors in that environment (male tsetse also feed exclusively on vertebrate blood). One way to overcome this obstacle is to release sterilized males that present enhanced refractoriness to parasite infection. This outcome is currently achieved by feeding the sterilized males twice with the drug isometamidium chloride prior to their release [70]. However, treated flies are not 100% resistant to infection [70], and the risk exists that the parasite will eventually develop resistance to the drug. Our data presented herein indicate that inoculating sterilized males with *Kco_Z* prior to their release would serve as an alternative, or supplemental, means of making the flies resistant to infection. Specifically, *Gmm*^WT/*Kco_Z*^ males (and females) are significantly more refractory to infection with trypanosomes than are their wild-type counterparts. This finding implies that sterilized, *Gmm*^WT/*Kco_Z*^ individuals would be relatively poor vectors of disease-causing trypanosomes and thus safer to release than sterilized males that do not house this bacterium. Furthermore, our preliminary analyses suggest that *Kco_Z* infection does not compromise the mating competitiveness nor sperm abundance of male tsetse, thus suggesting that *Gmm*^WT/*Kco_Z*^ individuals would be as successful as their wild counterparts at locating females and engaging in viable matings. Finally, male tsetse could be irradiated as pupae or teneral adults [71], prior to colonization with *Kco_Z*, thus eliminating the possibility that this treatment could detrimentally impact the bacterium’s fitness and thus its effect on fly vector competency. These characteristics provide preliminary evidence that releasing sterilized, *Kco_Z* infected male tsetse as part of an AW-IPM program would significantly reduce the capacity of these flies to transmit disease.

In conclusion, data presented in this study indicates that *Kco_Z* could effectively complement currently used AW-IPM programs aimed at reducing or eliminating tsetse populations by inhibiting trypanosome infection establishment in the fly’s gut. However, the complex relationship between tsetse, its indigenous (endosymbionts) and exogenous (trypanosomes and environmentally acquired microorganisms) microbiota, and *Kco_Z* must be studied in more detail before the bacterium is used in this capacity. Of particular importance are studies aimed at determining whether *Kco_Z* presents trypanocidal activity in other epidemiologically important tsetse species (e.g., *G*. *fuscipes*). Furthermore, field-based studies would shed light on how the ecology of tsetse’s natural environment influences the overall efficacy of the system.

## Materials and methods

### Ethical consideration

This work was carried out in strict accordance with the recommendations in the Office of Laboratory Animal Welfare at the National Institutes of Health and the Yale University Institutional Animal Care and Use Committee. The experimental protocol was reviewed and approved by the Yale University Institutional Animal Care and Use Committee (Protocol 2011–07266).

### Tsetse, bacteria and trypanosomes

Tsetse flies (*Glossina morsitans morsitans*) were maintained in Yale University’s insectary at 24°C with 55% relative humidity. Flies received defibrinated (via mechanical agitation for 20 minutes) bovine blood (Quad Five, Ryegate, MT, USA) through an artificial membrane feeding system every 48 h [72]. Aposymbiotic tsetse (*Gmm*^Apo^) were generated and maintained as described previously [73]. Throughout the manuscript, flies referred to as ‘teneral’ were unfed adults recently eclosed (≤ 24h) from their pupal case. All tsetse lines used in this study are described in S1 Table.

*Sodalis* were isolated from tsetse pupae as described previously [74], and subsequently maintained in liquid brain heart infusion (BHI) media (Becton Dickinson). When necessary, *Sodalis* were plated on either Brain Heart Infusion agar supplemented with 10% defibrinated bovine blood (BHIB) or Mitsuhashi-Maramorosch (MM)-agar plates. *Kosakonia cowanii* Zambiae [previously designated *Enterobacter* sp Zambiae (*Esp_Z*); GenBank accession number CP022690.1; 28], isolated from the gut of the mosquito, *Anopheles gambiae* [25]), and *Serratia marcescens* (strain db11; isolated from a moribund *Drosophila* sp.) [75] were grown in liquid LB media or on LB-agar plates at 30°C.

*In vivo Trypanosoma congolense* and *T*. *brucei brucei* (YTAT 1.1) were expanded in rats and harvested from infected blood at peak parasitemia. Rat blood containing blood stream form (BSF) parasites was aliquoted and cryopreserved (in the liquid nitrogen vapor phase, approximately -150°C) for subsequent tsetse challenge experiments.

### Recombinant PGRP-LB antibacterial assays

Antibacterial activity of recombinant (rec) PGRP-LB was determined as described previously [15], with minor modification. Specifically, recPGRP-LB was added (10 μg/ml of media) to early log-phase (OD = 0.2–0.4) cultures of *Kco_Z*, *Sodalis* and *E*. *coli*. Controls consisted of bacterial cultures exposed to bovine serum albumin. Using a plate-based quantification assay [58], *E*. *coli* and *Kco_Z* load was subsequently measured 1 hr. later, while *Sodalis* load was measured 24 hr. later. Results are presented as % of initial inoculum, which was determined by dividing the number of bacterial CFU present after treatment with recPGRP-LB by the number of CFU present prior to inoculation.

### Microbial infection assays

*Per os* bacterial challenge of wild-type (*Gmm*^WT^) and *Gmm*^Apo^ flies was performed by feeding teneral adults a heat inactivated (HI; 56**°**C for 1 hr) blood meal (to inactivate the vertebrate complement system) inoculated with 5x10^4^ colony forming units (CFU) of each bacterial strain per ml of blood. Because tsetse flies consume approximately 20 μl of blood per feeding, each fly is inoculated with 1x10^3^ bacterial cells. *Gmm*^Apo^ flies colonized with either *Sodalis* or *Kco_Z* are designated *Gmm*^Apo/*Sgm*^ and *Gmm*^Apo/*Kco_Z*^, respectively, and *Gmm*^WT^ flies colonized with *Kco_Z* are designated *Gmm*^WT/*Kco_Z*^. For all experiments that employed tsetse flies inoculated with either *Sodalis* or *Kco_Z*, bacterial midgut load was determined by homogenizing microscopically dissected gut tissue in 0.85% NaCl and serially diluting and plating the samples on LB-agar (*E*. *coli*, *Kco_Z* and *Serratia*) or BHIB or MM (*Sodalis*) plates supplemented with antibiotics [58]. CFU per plate were counted manually, and counts are presented in the corresponding Results subsections.

For trypanosome infections, all flies received infectious blood meals containing 1x10^6^/mL BSF *T*. *congolense* or *T*. *b*. *brucei* parasites. *Gmm*^Apo^, *Gmm*^Apo/*Sgm*^ and *Gmm*^Apo/*Kco_Z*^ were challenged as eight day old adults (3^rd^ blood meal), while *Gmm*^WT^ and *Gmm*^WT/*Kco_Z*^ flies were challenged as five day old adults (2^nd^ blood meals). For *Kco_Z*/trypanosome co-infection experiments, distinct groups of mature *Gmm*^WT^ individuals were inoculated with 1x10^6^/mL BSF *T*. *congolense* parasites and 5x10^4^ CFU/ml of *Kco_Z*. Two weeks post-trypanosome challenge, all flies were dissected and their midguts microscopically examined to determine parasite infection status.

### Detection and inhibition of tsetse reactive oxygen intermediates

*Kco_Z* and *Sodalis* cultures were grown to mid-log phase (OD = 0.25), and cell-free supernatants were generated via centrifugation. Hydrogen peroxide (H_2_O_2_) concentrations in bacterial culture supernatants were determined using an Amplex Red Hydrogen Peroxide/Peroxidase assay kit according to the manufacturer’s (Invitrogen) protocol. In brief, supernatants were incubated for 30 min. with the assay reagent, and resulting fluorescence units were quantified using a Bio-Tek plate reader.

Antioxidants were used to inhibit tsetse ROI activity *in vivo*. The assay used was similar to those described previously [18,29,30]. In brief, treated flies were offered a blood meal inoculated with trypanosomes [1x10^6^/mL BSF *T*. *b*. *brucei* (YTAT 1.1)] and supplemented with vitamin C (10mM) or cysteine (10μM). All subsequent meals also contained antioxidant supplements.

### Determination of bacterial acid production *in vitro*

*Sodalis* and *Kco_Z* were grown in their respective liquid media to an O.D. of 1.0. Subsequently, 5x10^6^ cells (this value represents the approximate maximum load to which these bacteria grow in tsetse’s gut; see Fig 1A) were diluted to a volume of 1 ml (again in respective liquid media) and heat-killed (80**°**C for 1.5 hr). Conditioned media containing dead cells was added to early log growth phase *T*. *b*. *brucei* YTAT 1.1 grown in a Beck’s medium (GE Hyclone), which contains phenol red. When in solution this compound serves as a pH-sensitive colorimetric indicator that changes from pink-red to yellow as environmental pH drops. Other treatment groups were inoculated with 1 ml of heated, clean LB (*Kco_Z* growth medium) or clean BHI (*Sodalis* growth medium), while the control group consisted of trypanosomes alone. Two hours after exposing *T*. *b*. *brucei* to treatment conditions, cultures were assayed to determine pH using a Mettler Toledo pH meter. The pH of trypanosome containing Beck’s medium was experimentally reduced (to pH 5.8) via the addition of 0.1N HCl, while HK *Kco_Z* extracts were buffered to pH 7.2 via the addition of 0.1N NaOH. Trypanosome load in all treatment and control groups was determined at 2, 5 and 24 hour time points by counting live parasites using a Brite-Line hemocytometer.

### Determination of bacterial acid production *in vivo*

Microbial regulation of pH in tsetse’s midgut was determined by feeding teneral *Gmm*^Apo^ flies a HI blood meal inoculated with either *Sodalis* or *Kco_Z* (5x10^4^ CFU/ml of blood). Additionally, teneral *Gmm*^WT^ flies received the same quantity of *Kco_Z*. Five days post-bacterial challenge, colonized individuals were administered a meal composed of sucrose (2.5%) and phenol red (0.04%) solubilized in water. Twenty-four hours later, the color of the solution contained in the midgut was determined by incising the fly abdomen and observing the intact gut using a dissecting microscope (Zeiss Discovery) equipped with a digital camera (Zeiss AxioCam MRc 5). Remaining flies were dissected and their guts were harvested, homogenized in 0.85% NaCl, serially diluted and plated onto MM-agar plates (prepared as described in [56]) supplemented with phenol red (0.025 g/L) and sucrose (a 2.5% sucrose solution was spread onto plates immediately prior to applying tsetse gut extracts). CFU per plate was counted manually, and the growth medium was monitored to observe pH-induced changes in color.

### Fitness assays

For all fitness assays, *Gmm*^WT^ teneral females and males were infected with *Kco_Z* during their first blood meal. To determine midgut weight, midguts were dissected from 9 day old *Gmm*^WT/*Kco_Z*^ and *Gmm*^WT^ females and males (24 h after consuming their last blood meal) and weighed using a Mettler Toledo (AL104) balance. The effect of *Kco_Z* infection on female fecundity was measured by quantifying the length of three gonotrophic cycles (GC) and by weighing pupal offspring. To measure GC length, *Gmm*^WT/*Kco_Z*^ and *Gmm*^WT^ females were mated as 5 day old adults and thereafter maintained in individual cages. All females were monitored daily to determine when they deposited larvae, and all deposited larvae were weighed.

The effect of *Kco_Z* infection on male reproductive fitness was measured by quantifying the mating competitiveness and sperm abundance of individuals that housed the bacterium versus those that did not. Mating competitiveness assays were performed in individual tubular cages (height, 6 cm; diameter 12.7 cm), each of which housed one 5 day old virgin female (fed twice). Subsequently, one age-matched *Gmm*^WT/*Kco_Z*^ and *Gmm*^WT^ male (also fed twice) was added to each cage. These males were distinguished from one another by removing the proximal tarsus of the right foreleg from one of the individuals. The arena was observed until one of the males had successfully mounted the female, at which point the cage was submerged in ice and the free male identified. To eliminate any bias associated with removal of the foreleg tarsus, the experiment was repeated twice (*n* = 40 cages per experiment), each time with either *Gmm*^WT/*Kco_Z*^ or *Gmm*^WT^ males receiving the distinguishing procedure. Sperm abundance was measured by RT-qPCR quantification of *sperm-specific dynein intermediate chain* (*sdic*) expression in the reproductive tracts of three and 14 day old (fed twice) virgin *Gmm*^WT/*Kco_Z*^ and *Gmm*^WT^ males. Absolute *sdic* transcript abundance was determined by comparing experimental sample cycle threshold (C_t_) values to those derived from an *sdic* internal standard curve.

*Sodalis fliC* and *Wigglesworthia thiC* gene specific primers were used to quantify the absolute abundance of these bacteria. This was performed by comparing *Sodalis fliC* and *Wigglesworthia thiC* cycle threshold (C_t_) values in *Gmm*^WT/*Kco_Z*^ and *Gmm*^WT^ females and males to those derived from symbiont gene-specific internal standard curves. Because *Wigglesworthia* and *Sodalis* can be polyploid [76,77], we normalized symbiont genome copy number to constitutively expressed tsetse *gapdh* copy number. All RT-qPCR primers are listed in S2 Table. All RT-qPCR assays were carried out in duplicate, and replicates were averaged for each sample. Negative controls were included in all amplification reactions.

### Statistical analyses

For trypanosome infection experiments, statistical analyses were carried out using the R software for macOS (version 3.3.2) or GraphPad Prism (v.6). A generalized linear model (GLM) was generated using binomial distribution with a logit transformation of the data. The binary infection status (infected or recovered) was analyzed as a function of the bacterium used to colonize the insects (or its absence). For experiments requiring a pairwise comparison, we performed a Wald test on the individual regression parameter (nature of the bacterium used to colonize) to test its statistical difference. For experiments requiring multiple comparisons, multiple pairwise tests were generated using Tukey contrasts on the generalized linear model (GLM) using glht function of "multcomp" package in R. For the mating assay, under the null hypothesis "*Kco_Z* does not change the attractiveness of the male", the probability that a female will chose either of the males is *p* = 0.5. We tested the validity of the null hypothesis by using a Chi-square goodness of fit test. Details of the statistical tests described above are indicated in S1 Dataset. All statistical tests used, and statistical significance between treatments, and treatments and controls, are indicated on the figures or in their corresponding legends. All samples sizes are provided in corresponding figure legends or are indicated graphically as points on dot plots. Biological replication implies distinct groups of flies were collected on different days, and where applicable, treated with distinct treatments (e.g., different bacterial cultures or individual aliquots of trypanosomes).

## Supporting information

S1 Fig Load [colony forming units (CFU) per fly gut] of exogenous *Kco_Z* and *Sodalis* in the gut of experimental flies, and *Kco_Z* acid production *in vitro*. *Kco_Z* and *Sodalis* CFU/gut in 8 day old *Gmm*^Apo/*Kco_Z*^ (*Kco_Z*) and *Gmm*^Apo/*Sgm*^ (*Sodalis*) flies prior to (A) challenge with 1x10^6^ blood stream form (BSF) trypanosomes per ml of blood and (B) measuring gut pH *in vivo*. (C) Guts from *Gmm*^Apo/*Kco_Z*^ (*Kco_Z*) and *Gmm*^Apo/*Sgm*^ (*Sodalis*) flies homogenized and plated onto MM-agar plates supplemented with phenol red (0.025 g/L) and sucrose (a 2.5% sucrose solution was spread onto plates immediately prior to applying gut extracts). Plate color reflects bacteria induced changes in pH relative to the empty control. (D) *Kco_Z* density in the gut of *Gmm*^WT/*Kco_Z*^ (*Kco_Z*) flies prior to measuring gut pH *in vivo*. (E) *Kco_Z* density in the gut of trypanosome infected (*Kco_Z* TI) and trypanosome refractory (*Kco_Z* TR) *Gmm*^WT/*Kco_Z*^ flies. Measurements were taken at the time infection status was determined (14 days post-challenge). (F) *Kco_Z* density in the gut of a random sample of *Gmm*^WT/*Kco_Z*^ (*Kco_Z*) flies used to determine the bacterium’s impact of tsetse fitness parameters. In panels (A), (B) and (D-F) bacterial load (CFU/gut) was determined via a plating assay described in the Materials and Methods (subsection *Microbial infection assays*) and in reference [58].
(TIF)

S1 Table Tsetse cohorts used in this study, and the status of their enteric microbiota. (XLSX)

S2 Table RT-qPCR primers used in this study. (XLSX)

S1 Dataset Statistical tests, and their results, used in this study. (XLSX)
