# Correction: Colonization of the tsetse fly midgut with commensal *Kosakonia cowanii* Zambiae inhibits trypanosome infection establishment

**DOI:** 10.1371/journal.ppat.1007688

**Published:** 2019-04-08

**Authors:** Brian L. Weiss, Michele A. Maltz, Aurélien Vigneron, Yineng Wu, Katharine S. Walter, Michelle B. O’Neill, Jingwen Wang, Serap Aksoy

The affiliation for the second author is incorrect. Michele Maltz is not affiliated with #2 but with #1: Yale School of Public Health, Department of Epidemiology of Microbial Diseases, New Haven, Connecticut, United States of America.

Additionally, the current address for Michele Maltz is: Southern Connecticut State University, New Haven, Connecticut, United States of America.
