# Supplementary figures and images for: Colonization of the tsetse fly midgut with commensal Kosakonia cowanii Zambiae inhibits trypanosome infection establishment
Source: PLoS Pathog. 2019 Feb 28;15(2):e1007470. doi: 10.1371/journal.ppat.1007470 (PMC6394900; doi:10.1371/journal.ppat.1007470)

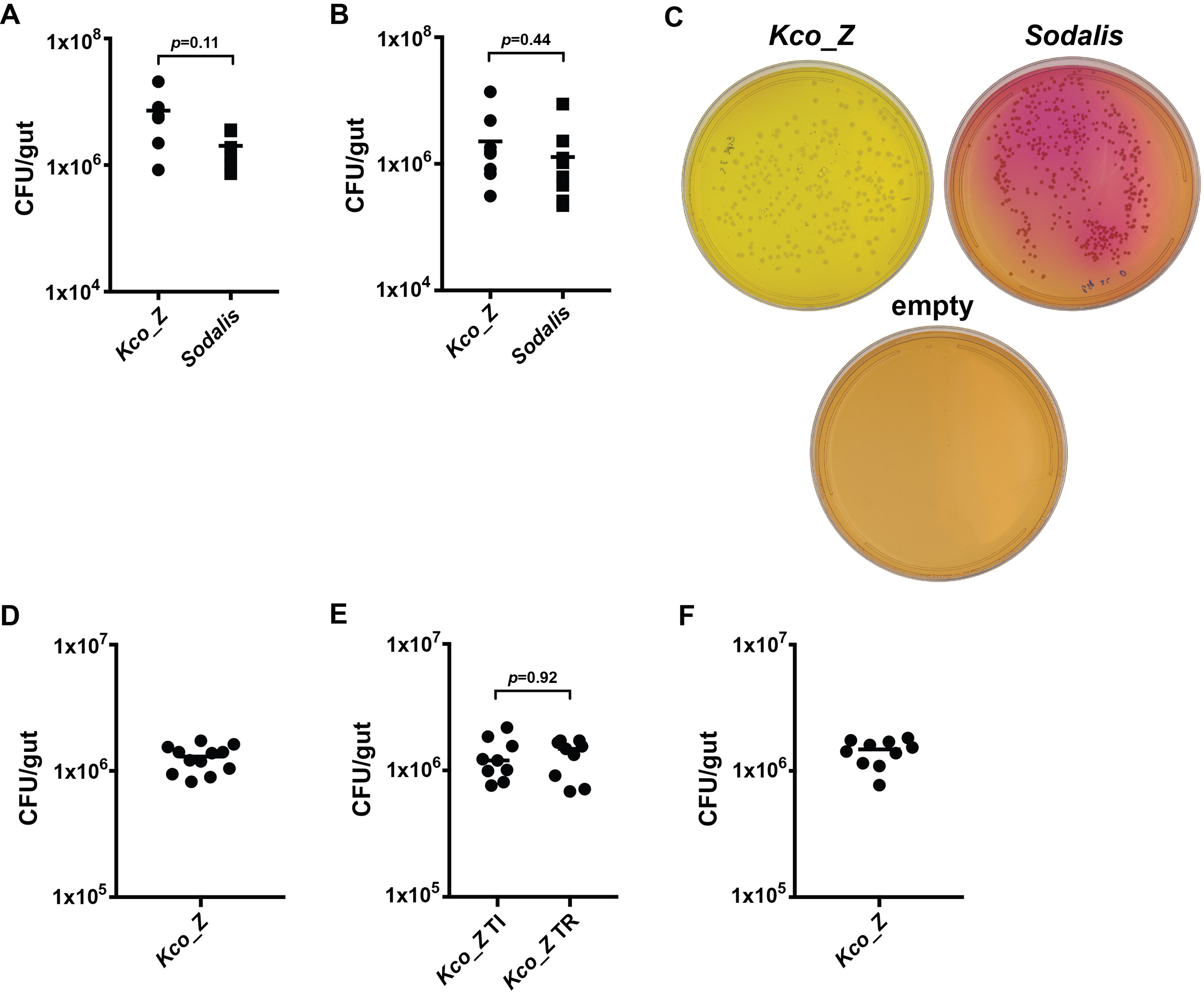

Supplement: S1 Fig — Kco_Z and Sodalis CFU/gut in 8 day old GmmApo/Kco_Z (Kco_Z) and GmmApo/Sgm (Sodalis) flies prior to (A) challenge with 1x10^6^ blood stream form (BSF) trypanosomes per ml of blood and (B) measuring gut pH in vivo. (C) Guts from GmmApo/Kco_Z (Kco_Z) and GmmApo/Sgm (Sodalis) flies homogenized and plated onto MM-agar plates supplemented with phenol red (0.025 g/L) and sucrose (a 2.5% sucrose solution was spread onto plates immediately prior to applying gut extracts). Plate color reflects bacteria induced changes in pH relative to the empty control. (D) Kco_Z density in the gut of GmmWT/Kco_Z (Kco_Z) flies prior to measuring gut pH in vivo. (E) Kco_Z density in the gut of trypanosome infected (Kco_Z TI) and trypanosome refractory (Kco_Z TR) GmmWT/Kco_Z flies. Measurements were taken at the time infection status was determined (14 days post-challenge). (F) Kco_Z density in the gut of a random sample of GmmWT/Kco_Z (Kco_Z) flies used to determine the bacterium’s impact of tsetse fitness parameters. In panels (A), (B) and (D-F) bacterial load (CFU/gut) was determined via a plating assay described in the Materials and Methods (subsection Microbial infection assays) and in reference [58]. (TIF) [file ppat.1007470.s001.tif]
